# Silk Spinning Deficiency Causes Delayed Fat Body Dissociation Accompanied by Lipid Metabolic Reprogramming in *Bombyx mori*

**DOI:** 10.3390/biology15212025

**Published:** 2026-11-06

**Authors:** Ruji Peng, Xiaoning Sun, Cheng Luo, Jianghan Du, Yanhong Jiang, Yanghu Sima, Guang Wang, Shiqing Xu

**Affiliations:** 1 School of Life Sciences, Suzhou Medical College, Soochow University, Suzhou 215123, China; 2 Institute of Agricultural Biotechnology & Ecology (IABE), Soochow University, Suzhou 215123, China

**Keywords:** *Bombyx mori*, silk spinning deficiency, fat body, dissociation, lipid metabolic reprogramming

## Abstract

Silk spinning deficiency is often associated with developmental abnormalities and diminished vitality. In silkworm, the fat body functions as a crucial organ in nutrient metabolism and metamorphic development. However, the impact of silk spinning deficiency on fat body development and metabolic function during metamorphosis remains unclear. Here, using a silkworm model of silk spinning deficiency, we systematically analyzed its effects on fat body regeneration and metabolism during metamorphosis by integrating morphological observations, transcriptomics, untargeted metabolomics, histological staining, and biochemical and molecular assays. Silk spinning deficiency delayed the onset of fat body dissociation and impeded its subsequent remodeling, accompanied by extensive transcriptional reprogramming of metabolism-related genes in the fat body. Notably, the glucose metabolism pathway, a major energy source, shifted toward glycolysis and lactate production. Untargeted metabolomic analysis revealed marked perturbations in the metabolic networks of amino acids and lipids in the fat body. Lipid content analysis showed that silk spinning deficiency promoted the accumulation of lipid droplets and triglycerides in the fat body, potentially linked with the activation of the IIS/TOR signaling pathway. Collectively, these results demonstrate that silk spinning deficiency disrupts nutrient utilization and allocation during metamorphosis, thereby interfering with fat body dissociation and remodeling while promoting lipid accumulation.

## Simple Summary

Silkworms spin silk before pupation, and their fat body is the central organ for nutrient metabolism and undergoes tissue remodeling during metamorphosis. It remains unclear whether loss of normal silk spinning affects fat body development and metabolism. In this study, we used a silkworm model of defective silk spinning to examine fat body changes. We found that this defect delayed the onset of fat body dissociation and blocked the completion of its remodeling. It also triggered widespread metabolic alterations in the fat body. Integrated transcriptomic and metabolomic data indicate a shift in carbohydrate metabolism toward glycolysis and lactate production, together with perturbed amino acid and lipid metabolism. Defective silk spinning increased lipid storage within the fat body, which may be linked to activation of nutrient-sensing signaling. Our results suggest that defective silk spinning disrupts nutrient utilization and allocation during metamorphosis, thereby interfering with fat body dissociation and remodeling and promoting lipid accumulation. This study provides new insight into the interplay among silk gland function, fat body remodeling, and metabolic regulation during insect metamorphosis.

## 1. Introduction

The silkworm, *Bombyx mori*, is a typical silk-secreting insect and an important lepidopteran model for studying holometabolous development [1]. During the fifth larval instar, the highly specialized silk glands of silkworm synthesize large amounts of silk proteins, which are subsequently secreted them externally during the spinning stage [2,3]. After completion of spinning, the silk glands rapidly degenerate, and luminal remnants and tissue components are degraded or contribute to material recycling during metamorphosis [3,4,5]. Thus, silk spinning physiology may influence the development and metabolic status of other tissues during metamorphosis. Silk spinning deficiency is frequently observed in silkworm rearing practices and silk gland gene editing [6,7,8]. This deficiency is caused by multiple factors, including silk gland development, silk protein synthesis and secretion, and impeded spinning, leading to the formation of thin cocoons or even naked pupae, with large amounts of silk proteins retained in the body and varying degrees of difficulty in metamorphic development [9,10,11]. Existing studies have predominantly focused on silk gland degeneration and endocrine dysfunctions [12,13,14]; however, the effects on tissue remodeling and metabolism during metamorphosis remain unclear.

In insects, the fat body functions as the primary organ for nutrient storage and intermediary metabolism, significantly contributing to the storage, conversion, and mobilization of nutrients such as glycogen, lipids, and amino acids [15,16,17]. The fat body also serves as a typical tissue-remodeling organ during metamorphosis and pupation in the silkworm [18], exhibiting stage-specific morphological and functional changes during metamorphic development [19,20]. Previous studies have confirmed notable cross-tissue metabolic interaction between the silk gland and fat body [21,22]. Nutrients mobilized from the larval fat body can be delivered via the hemolymph to the silk gland to support silk protein synthesis [23,24], whereas some macromolecules from degenerating silk glands during metamorphosis can be transferred to the fat body as substrates [5,25]. Silk gland-specific functional perturbation can alter multiple metabolic pathways in the fat body [22], and an abnormal metabolic environment in the hemolymph can affect nutrient storage and sensing as welas metamorphic remodeling of the fat body [19,26]. Moreover, in non-cocoon-producing silkworms obtained by inhibiting silk protein synthesis, amino acids that constitute silk proteins accumulate in the hemolymph, lipid and carbohydrate reserves in the pupal fat body remain intact, and the IIS/TOR nutrient-sensing signaling pathway is enhanced [21]. These findings suggest that silk spinning deficiency may substantially influence the and functionality of the fat body during metamorphosis.

The silkworm exclusively feeds only during its larval stage. During pupal metamorphosis, fat body remodeling does not merely reflect a morphological change in tissue architecture; rather, it entails the synchronized mobilization of nutrient reserves and reprogramming of metabolic processes, signifying a pivotal transition in the fat body’s primary physiological functions and metabolic modes, shifting from nutrient synthesis and storage to energy supply [27,28]. During this process, the storage, mobilization, and utilization of nutrients such as amino acids, carbohydrates, and lipids change markedly and are jointly regulated by mechanisms of nutrient sensing and endocrine signaling [16,29]. Time-course transcriptomic analysis has revealed stage-specific variations in the expression of genes related to lipid synthesis and degradation during metamorphosis and pupation, and the disruption of BMP signaling can simultaneously affect fat body dissociation and lipid mobilization [19], indicating that fat body remodeling is closely associated with metabolic regulation in the silkworm. Hence, it is necessary to investigate the developmental and metabolic changes in the fat body following a shift in nutrient allocation due to silk spinning deficiency.

Accordingly, this study used a silkworm model with normal silk gland development and silk protein synthesis but exhibiting silk spinning deficiency to analyze the effects of this silk spinning deficiency on fat body dissociation and remodeling during metamorphosis. By conducting transcriptome sequencing and untargeted metabolomics, we dissected the molecular responses of the fat body at the transcriptional and metabolic levels. This study aimed to clarify the link between abnormal silk spinning and fat body remodeling during metamorphosis, providing new evidence for understanding the coordination of fat body development and metabolism during silkworm metamorphosis.

## 2. Materials and Methods

### 2.1. Animal Preparation

Larvae of the Dazao strain of *Bombyx mori* were reared throughout the generation at 25 °C, 75% relative humidity, and a 12 h light/dark cycle, and were fed fresh mulberry leaves. The silkworm model of silk spinning deficiency was established according to a previously described method [18,30,31]. Female larvae at the wandering stage were selected, and their spinnerets were sealed with 55–60 °C liquid paraffin to prevent spinning, thereby generating the silk spinning deficiency animal model (AM); normal spinning silkworms were used as the control group (CK) (Figure S1A,B). Individuals in both groups were maintained under identical conditions. At predetermined time points after modeling (0, 24, 48, 72, 96, 120, 144, 168, and 192 h), larvae or pupae were surface-sterilized with 75% ethanol, fixed on disinfected paraffin plates, and dissected to obtain the fat body and silk gland.

### 2.2. Quantitative Reverse Transcription Polymerase Chain Reaction (qRT-PCR)

Total RNA was extracted from the fat body using TRIzol^®^ Reagent (15596018, Invitrogen™, Carlsbad, CA, USA). RNA was reverse-transcribed into cDNA using a reverse transcription kit (RR037B; TaKaRa, Dalian, China). qRT-PCR was performed on an ABI StepOnePlus™ real-time PCR system (Ambion, Foster City, CA, USA) in a 20 μL reaction volume. Three independent biological replicates were analyzed for each sample. Primers were designed using the NCBI Primer designing tool (https://www.ncbi.nlm.nih.gov/tools/primer-blast/index.cgi?LINK_LOC=BlastHome) (accessed on 6 March 2026), and those used for qRT-PCR are listed in Table S1.

### 2.3. RNA-Seq and Bioinformatics Analysis

Fat body samples were collected from CK and AM silkworms at 48 and 96 h after modeling, and ground into powder in liquid nitrogen. Each group contained three replicates, each consisting of equal amounts of fat body pooled from three individuals. Total RNA was extracted from the powdered fat body samples using TRIzol^®^ Reagent (15596018, Invitrogen™, Carlsbad, CA, USA). Transcriptome library construction and sequencing were performed by Shanghai Majorbio Bio-Pharm Technology Co., Ltd. (Shanghai, China) on the Illumina NovaSeq 6000 platform. Reads were aligned to the *Bombyx mori* genome (https://www.ncbi.nlm.nih.gov/genome/?term=Bombyx+mori) (accessed on 17 June 2025) using HISAT2 (Version 2.2.1). Expression levels of genes and novel transcripts were estimated using transcripts per million (TPM). Differentially expressed genes (DEGs) were identified with |log2 (fold change)| ≥ 1 and q-value < 0.05 as the significance threshold. Gene expression analysis was performed based on log10 (TPM + 1) values. DEGs were annotated and subjected to enrichment analysis using the GO and KEGG databases.

### 2.4. Untargeted Metabolomics

Fat body samples were collected from CK and AM silkworms at 48 and 96 h after modeling. Each group contained five replicates, each consisting of equal amounts of fat body pooled from three individuals. A 400 μL volume of methanol solution (methanol: water = 4:1, *v*/*v*) was added to the fat body tissue. The tissue was homogenized at low temperature using a high-throughput tissue homogenizer. After vortexing, the samples were ultrasonicated on ice for extraction three times, 10 min each. The samples were then kept at −20 °C for 30 min and centrifuged at 13,000× *g* for 15 min at 4 °C, after which the supernatant was collected. The supernatant was analyzed by Shanghai Majorbio Bio-Pharm Technology Co., Ltd. (Shanghai, China) using a UPLC-TripleTOF system. LC-MS data were imported into Progenesis QI (Version 3.0) (Nonlinear Dynamics, Waters, Milford, MA, USA) for metabolite annotation. The processed data were uploaded to the Majorbio Cloud Platform (https://cloud.majorbio.com) (accessed on 12 April 2026) for data analysis. Differential accumulation metabolites (DAMs) were screened using *p*-value ≤ 0.05 and VIP ≥ 1 as thresholds. The differential abundance score (DA Score) formula to calculate pathway up- and down-regulation. The formula is as follows: (number of upregulated metabolites − number of downregulated metabolites)/total number of metabolites annotated.

### 2.5. Green Fluorescence Detection of Lipid Droplets

A lipid droplet green fluorescence detection kit (BDPY 493/503, C2053S, Beyotime, Nantong, China) was used to detect lipid droplet abundance in the fat body. At 48 and 96 h after modeling, CK and AM silkworms were wiped with absolute ethanol and dissected on a sterile bench. Fat body tissues were excised with forceps, quickly transferred to insect physiological saline (0.7% NaCl), and freed of adherent impurities such as tracheae. The tissues were washed once more with insect physiological saline and then fixed in 4% paraformaldehyde for 60 min. After washing with PBS, the samples were incubated with BODIPY 505/515 working solution in the dark for 2 h, followed by three washes with PBS, 5 min each. The samples were then incubated with DAPI staining solution for 30 min, followed by three washes with PBS, 5 min each. Coverslips were mounted and sealed with transparent nail polish. Fluorescence images were captured using an Olympus BX51 microscope (Olympus, Tokyo, Japan). All samples were imaged under identical settings, and BODIPY fluorescence was measured using ImageJ software (ImageJ 2.9.0/1.53t; Java 1.8.0_322 [64-bit]). Three independent biological replicates were analyzed.

### 2.6. Triglyceride (TG) Detection

According to the reagent instruction (Amplex Red Triglyceride Assay Kit, S0219S, Beyotime, Nantong, China), after adding 200 μL of isopropanol to every 20 mg of tissue, use a glass homogenizer to homogenize at 4 °C, and then centrifuge the tissue homogenate at 12,000 r/min in a 4 °C precooled centrifuge for 5 min, and take the supernatant for subsequent detection. After diluting the supernatant 20 times with deionized water, add 25 μL of sample diluent to the 96-well plate, and set up the reference hole and standard curve hole according to the instructions. Add 2 μL lipase into each well, mix well, and incubate at 37 °C for 20 min. After incubation, add 50 μL of triglyceride detection working solution into each well, mix well, and then react in a 37 °C incubator for 60 min without light. Finally, the absorbance at A570 was detected by a microplate reader. The standard curve is established according to the calculation formula in the manual, and the triglyceride (TG) content in the fat body sample is calculated through the standard curve. Each group contained five replicates, each consisting of equal amounts of fat body pooled from three individuals.

### 2.7. Western Blotting

Total protein was extracted from fat body samples of CK and AM silkworms at 48 and 96 h after modeling. Proteins were separated by 10% SDS-PAGE and transferred to a PVDF membrane. Primary antibodies included anti-p-Akt (4060, Cell Signaling Technology, Danvers, MA, USA; 1:2,000, rabbit antibody), anti-Akt (4691, Cell Signaling Technology, Danvers, MA, USA; 1:1,000, rabbit antibody), anti-p-S6K (9234, Cell Signaling Technology, Danvers, MA, USA; 1:1,000, rabbit antibody), and anti-β-actin (ab52901, Abcam, Cambridge, UK; 1:1,000, mouse antibody). The secondary antibodies were HRP-labeled anti-rabbit IgG (BS20241-Y, BioWorld Technology, Minneapolis, MN, USA; 1:5,000) and goat anti-mouse IgG (BS20242-Y, BioWorld Technology, Minneapolis, MN, USA). Finally, the immunoblots were visualized using EZ-ECL chemiluminescence reagent (1705061, Bio-Rad, Hercules, CA, USA) and a ChemiDoc imaging system (1708370, Bio-Rad, Hercules, CA, USA). Image Lab (Bio-Rad, Hercules, CA, USA) (Version 5.2.1 build 11) was used to process and analyze the obtained images. Four independent biological replicates of p-S6K and p-Akt were analyzed, as were three independent biological replicates of t-Akt.

### 2.8. Data Analysis

Statistical analyses were performed using GraphPad Prism 8 (La Jolla, CA, USA) (Version 8.0.2). The multi-group comparison data on mRNA levels of fat body dissociation-related genes, statistical analysis of amino acid metabolism-related DEGs, statistical analysis of glucose metabolism-related DEGs, changes in amino acid content in the metabolomic, lipid droplet content in the fat body, TG content in the fat body, transcript levels of IIS/TOR pathway-related genes and Protein levels of insulin signaling pathway-related components were subjected to multiple comparison t-test and statistical significance determined using the Holm–Sidak method. Data are presented as the mean ± SEM. 

## 3. Results

### 3.1. Silk Spinning-Deficient Silkworms Exhibit Delayed Initiation of Fat Body Dissociation

**Figure 1:**
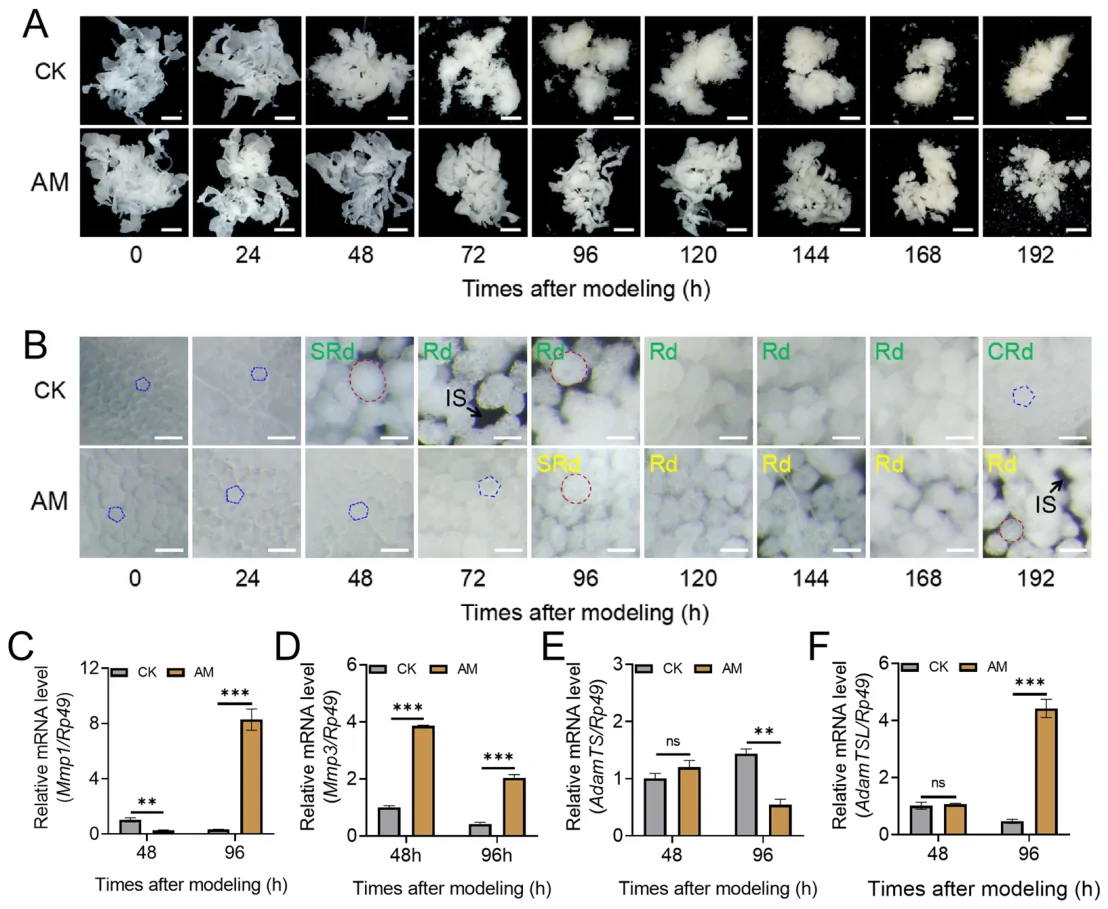
Fat body dissociation and remodeling during metamorphosis in silkworms with silk spinning deficiency. (**A**) Morphological changes in fat body tissue during development. Scale bar: 1000 μm. (**B**) Morphological changes in fat body cells during development. Scale bar: 50 μm. SRd, start of fat body remodeling. Rd, fat body remodeling process. CRd, completion of fat body remodeling. IS, intercellular space. Blue dashed lines indicate fat body cells before the initiation of remodeling and after completion of remodeling; red dashed lines indicate fat body cells at the initiation of remodeling. (**C***–***F**) mRNA levels of fat body dissociation-related genes. *Rp49* was used as the internal reference gene. N = 3. C, *Mmp1*, matrix metalloproteinase 1. D, *Mmp3*, matrix metalloproteinase 3. E, *AdamTS*, A disintegrin and metalloproteinase with thrombospondin motifs 1. F, *AdamTSL*, A disintegrin and metalloproteinase with thrombospondin motifs like. Significance levels are indicated as: **, *p* < 0.01; ***, *p* < 0.001; ns, *p* > 0.05.

During the spinning process, silk dope in the silk gland lumen is rapidly expelled. In the CK group, the silk glands rapidly atrophied and degenerated 48 h after the initiation of spinning and had completely disappeared by 120 h. Conversely, the AM group exhibited gradual swelling of the silk glands from 0 to 48 h after modeling, with degeneration commencing only after 72 h; the glands had not completely degenerated by 192 h (Figure S1C). Morphological analysis of the fat body during the same period showed that the larval fat bodies in the CK group, which were closely interconnected and organized in a single layer, began to dissociate into individual cells at 48 h after modeling, with cell edges transitioning from regular polygons to more rounded forms. By 96 h, the dissociation process was complete, and remodeling of the fat bodies had commenced; by 192 h, adult-type fat bodies with tight connections, regular cell edges, and multiple layers had formed. In contrast, the AM group showed markedly different morphological progression of fat body dissociation and remodeling. At 48 h post modeling, fat body cells remained tightly connected; irregular individual cells emerged only at 96 h, and complete dissociation was achieved by 192 h (Figure 1A,B). The analysis of the transcript levels of dissociation-related genes at key stages of fat body morphological divergence of fat bodies showed that, compared to the CK group, the transcript level of *Mmp1* was significantly downregulated and that of *Mmp3* was significantly upregulated in the AM group at 48 h after modeling, whereas *AdamTS* and *AdamTSL* transcript levels showed no differences. At 96 h post modeling, except fordownregulated *AdamTS* expression, the transcript levels of *Mmp1*, *Mmp3*, and *AdamTSL* in the AM group were significantly more than 5-fold higher than those in the CK group (Figure 1C–F). These results suggest that silk-spinning-deficient silkworms exhibit delayed initiation of fat body dissociation and remodeling process.

### 3.2. Silk Spinning Deficiency Causes Transcriptional Reprogramming of Fat Body Metabolism

**Figure 2:**
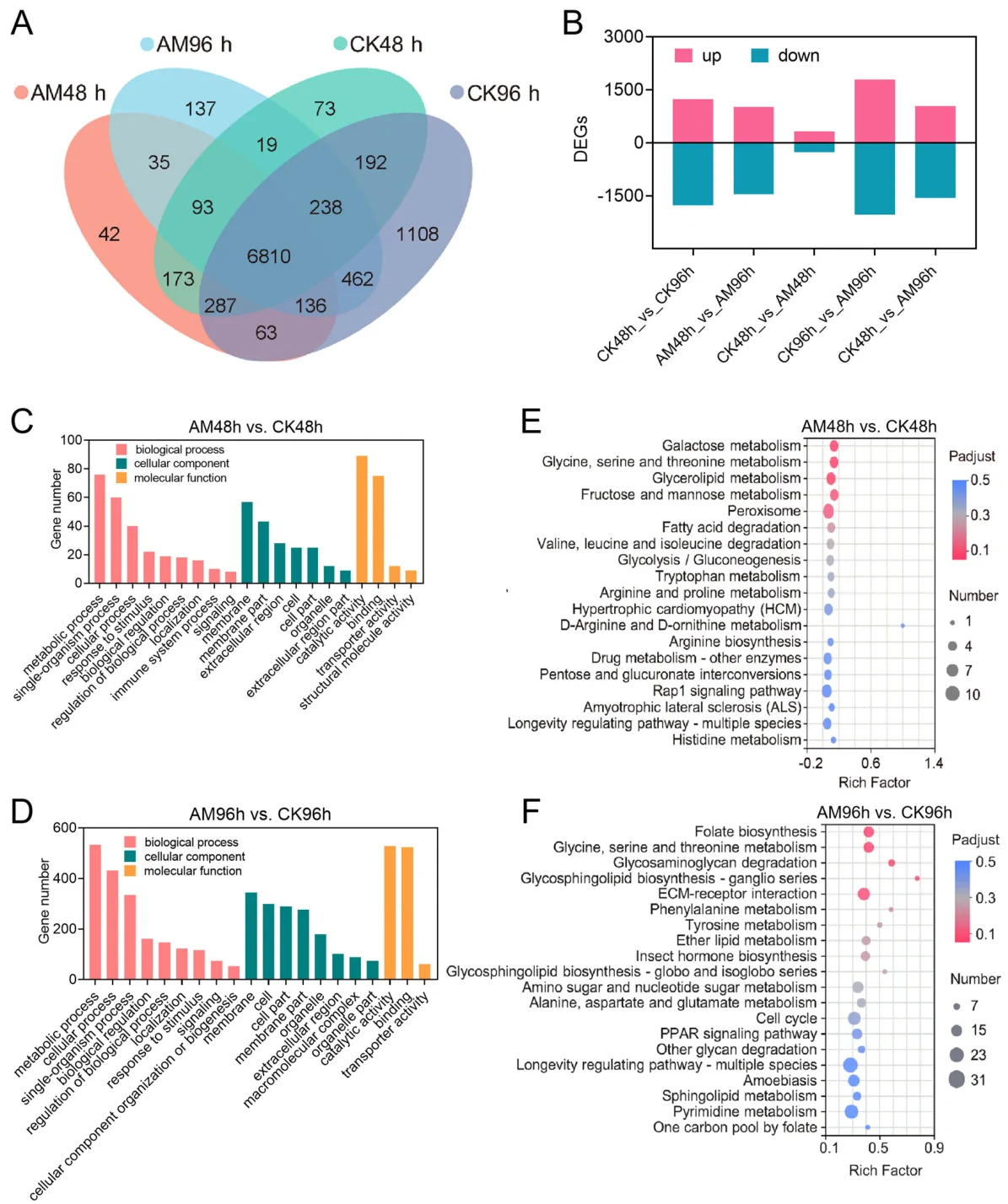
Transcriptomic sequencing of the fat body and enrichment analysis of differentially expressed genes. (**A**) Venn diagrams of common and unique genes expressed in different groups. (**B**) Numbers of DEGs. (**C**,**D**) GO annotation analysis of DEGs at 48 h (**C**) and 96 h (**D**) after modeling between AM and CK. (**E**,**F**) KEGG enrichment analysis of DEGs at 48 h (**E**) and 96 h (**F**) after modeling between AM and CK.

To investigate the effects of silk spinning deficiency on the genome-wide transcriptional profile of the fat body, transcriptome sequencing was performed on fat body samples from the CK and AM groups at 48 and 96 h after modeling (Figure 2A,B). Analysis of unique transcripts revealed greater between-group differences at 96 h (Figure 2A). Statistical analysis of DEGs showed that the CK96h group had 1238 upregulated and 1016 downregulated genes compared to the CK48h group, whereas the AM96h group had 1769 upregulated and 1463 downregulated genes compared to the AM48h group. At the same time points, 591 and 3824 DEGs were identified between the AM and CK groups at 48 and 96 h, respectively, indicating that prolonged silk spinning deficiency significantly altered gene expression in the fat body gene expression (Figure 2B). These results suggest that silk spinning deficiency significantly alters and exacerbates gene expression patterns in the fat body.

GO analysis revealed that the identified DEGs were mainly enriched in biological processes such as metabolic processes, cellular processes, and single-organism processes; cellular components mainly involved the membrane, cell, and extracellular region; and molecular functions were primarily catalytic activity and binding (Figure 2C,D), indicating that DEGs are extensively involved in metabolism.

KEGG pathway enrichment analysis showed that at 48 h after modeling, the identified DEGs were mainly enriched in core metabolic pathways such as carbohydrate metabolism, amino acid metabolism, and lipid metabolism (Figure 2E). At 96 h post modeling, the DEGs were more enriched in folate biosynthesis, glycosaminoglycan degradation, ECM-receptor interaction, insect hormone biosynthesis, cell cycle, PPAR signaling pathway, and various amino acid and lipid metabolic pathways (Figure 2F). These results suggest that silk spinning deficiency may affect developmental remodeling in the fat body by interfering with core metabolic networks. In addition, KEGG pathway enrichment analysis of CK48h and AM96h showed that DEGs were enriched in metabolic pathways of glycine, serine, and threonine (Figure S4).

**Figure 3:**
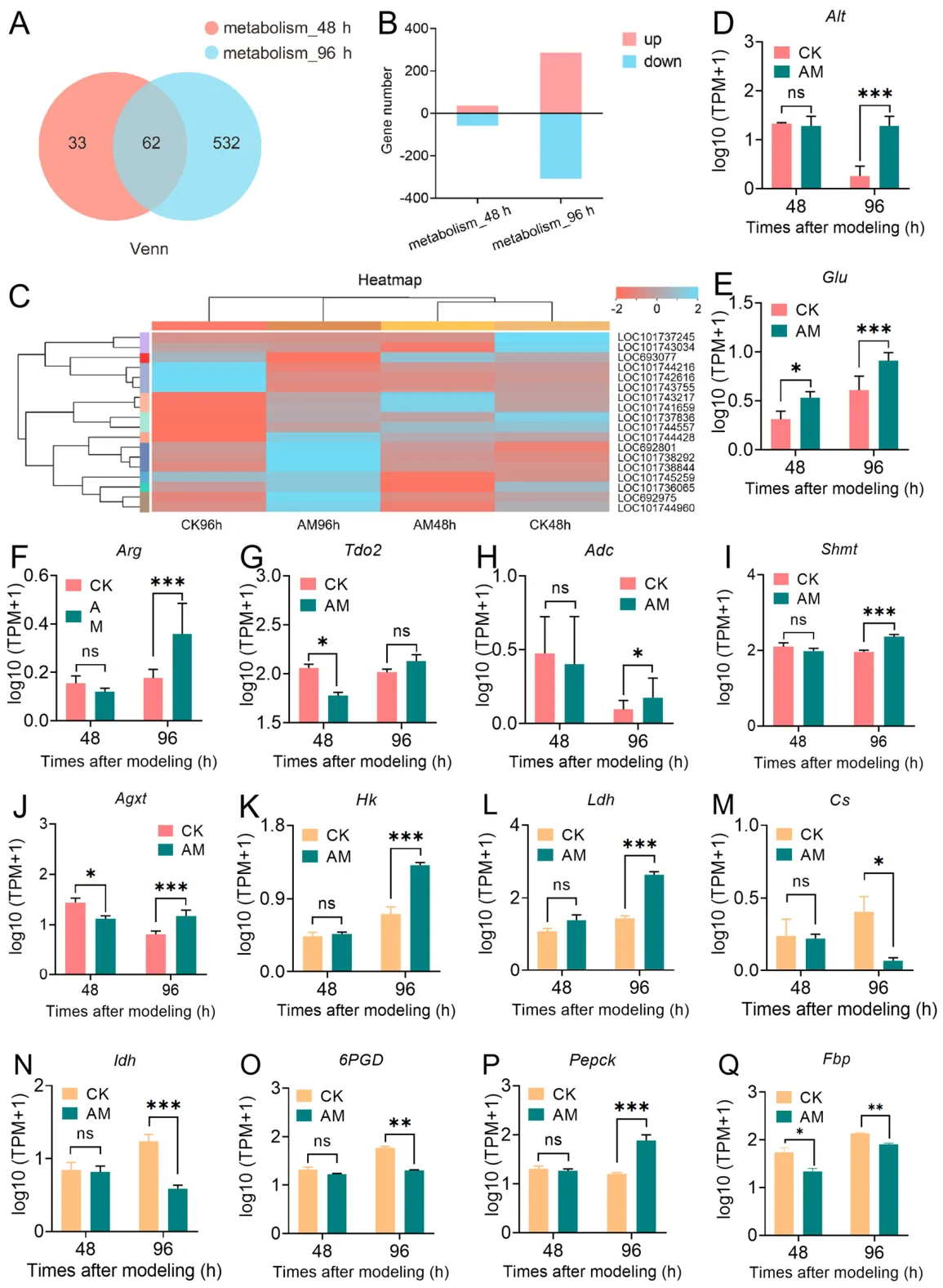
Analysis of DEGs involved in metabolic processes. (**A**) Venn diagram of metabolism-related DEGs. metabolism_48 h and metabolism_96 h represent the sets of metabolism-related DEGs in the CK and AM groups at 48 and 96 h after modeling, respectively. (**B**) Number of metabolism-related DEGs. (**C**) Clustering heatmap of 18 DEGs in the glycine, serine, and threonine metabolic pathways. (**D**–**J**) Statistical analysis of amino acid metabolism-related DEGs. N = 3. (**D**) *Alt*, alanine aminotransferase 1, catalyzes reversible transamination between alanine and α-ketoglutarate. (**E**) *Glu*, glutaminase, catalyzes the hydrolysis of glutamine to glutamate and free ammonia. (**F**) *Arg*, arginase, hydrolyzes L-arginine to L-ornithine and urea. (**G**) *Tdo2*, tryptophan 2,3-dioxygenase, the rate-limiting enzyme of tryptophan metabolism. (**H**) *Adc*, aspartate decarboxylase, catalyzes decarboxylation of L-aspartate to alanine. (**I**) *Shmt*, serine hydroxymethyltransferase, catalyzes the reversible conversion between serine and glycine. (**J**) *Agxt*, serine-pyruvate aminotransferase, catalyzes reversible transamination between L-serine and pyruvate. (**K**−**Q**) Statistical analysis of glucose metabolism-related DEGs. N = 3. (**K**) *Hk*, hexokinase type 2, the first key rate-limiting enzyme in glycolysis. (**L**) *Ldh*, lactate dehydrogenase, catalyzes the reversible conversion between pyruvate and lactate, accompanied by interconversion between NADH and NAD^+^. (**M**) *Cs*, Citrate synthase, a rate-limiting enzyme in the TCA cycle. (**N**) *Idh*, isocitrate dehydrogenase, catalyzes the oxidative decarboxylation of isocitrate to α-ketoglutarate. (**O**) *6PGD*, 6-phosphogluconate dehydrogenase, a key rate-limiting enzyme in the pentose phosphate pathway, catalyzes the oxidative decarboxylation of 6-phosphogluconate to ribulose-5-phosphate while reducing NADP^+^ to NADPH. (**P**) *Pepck*, mitochondrial phosphoenolpyruvate carboxykinase, a key hub enzyme linking the TCA cycle to gluconeogenesis/glycolysis. (**Q**) *Fbp*, fructose-1,6-bisphosphatase, a key rate-limiting enzyme in gluconeogenesis. Significance levels are indicated as: *, *p* < 0.05; **, *p* < 0.01; ***, *p* < 0.001; ns, *p* > 0.05.

Focusing on transcriptional differences in fat body metabolism-related genes, we statistically analyzed metabolism-related DEGs at 48 and 96 h after modeling. A total of 95 metabolism-related DEGs were identified at 48 h (37 upregulated and 58 downregulated), increasing to 594 at 96 h (286 upregulated and 308 downregulated), and there were 62 core metabolic genes that responded to silk spinning dificiency at 48 h and 96 h (Figure 3A,B). It is suggested that, as the modeling duration increases, the transcriptional changes in genes related to fat body metabolism are exacerbated by silk spinning deficiency, indicating that the metabolic response of the fat body exhibits temporal dynamics. A clustering heatmap of 18 DEGs in the metabolic pathways of glycine, serine, and threonine—the major amino acidsconstituting silk proteins—further showed that differences in gene expression levels were more pronounced at 96 h than at 48 h after modeling (Figure 3C).

In amino acid metabolism, compared to the CK group, the AM group exhibited significant upregulation of the transcript levels of degradation-related genes, including alanine aminotransferase 1 (*Alt*), glutaminase (*Glu*), arginase (*Arg*), tryptophan 2,3-dioxygenase (*Tdo2*), and aspartate decarboxylase (*Adc*), at 96 h after modeling (Figure 3D−H). Serine hydroxymethyltransferase (*Shmt*) expression in the AM group showed no significant difference from that in CK group at 48 h after modeling but was significantly upregulated at 96 h (Figure 3I), whereas the transcript level of serine-pyruvate aminotransferase *(Agxt*) first decreased and then increased (Figure 3J).

In glucose metabolism, compared to the CK group, the AM group showed a substantial increase in the transcript levels of the glycolytic rate-limiting enzymes hexokinase (*Hk*) and L-lactate dehydrogenase (*Ldh*) were significantly upregulated in the AM group at 96 h post modeling (Figure 3K−L), whereas the tricarboxylic acid (TCA) cycle rate-limiting enzymes citrate synthase (*Cs*) and isocitrate dehydrogenase (*Idh*) were significantly downregulated (Figure 3M,N); this findingindicates that pyruvate is preferentially converted to lactate rather than being utilized for energy production in the TCA cycle. The transcript level of 6-phosphogluconate dehydrogenase (*6PGD*), a rate-limiting enzyme of the pentose phosphate pathway, was significantly downregulated, possibly reducing NADPH production (Figure 3O). Phosphoenolpyruvate carboxykinase (*Pepck*), the initial rate-limiting enzyme of gluconeogenesis was upregulated, whereas the terminal key enzyme fructose-1,6-bisphosphatase (*Fbp*) was downregulated, suggesting that gluconeogenesis was inhibited in the AM group (Figure 3P,Q). These results indicate that silk spinning deficiency markedly alters the transcriptional expression patterns of genes related to amino acid catabolism, glycolysis, and gluconeogenesis in the fat body, suggesting possible reprogramming of the core metabolic network.

### 3.3. Silk Spinning Deficiency Induces Amino Acid and Lipid Metabolic Disorders and Excessive Lipid Accumulation in the Fat Body

**Figure 4:**
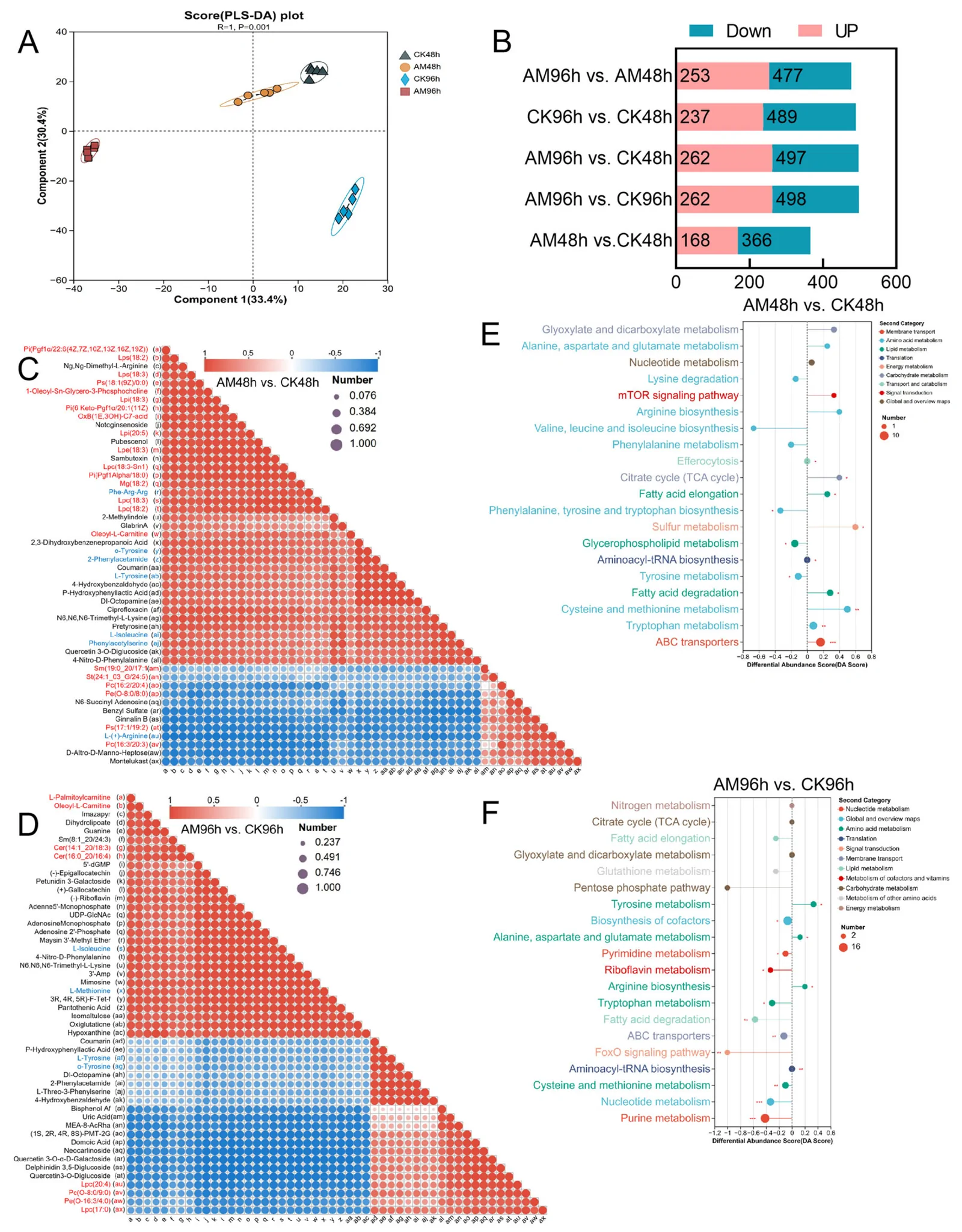
Metabolomic analysis of the fat body. (**A**) Partial least squares discriminant analysis (PLS-DA). (**B**) Statistics of DAMs. The horizontal axis indicates the number of differentially abundant metabolites, and the vertical axis indicates the names of the compared sample groups. (**C**,**D**) Correlation analysis of differential metabolites. Mg(18:2), linoleic acid; oleoyl-L-carnitine, a long-chain acylcarnitine and an intermediate of fatty acid β-oxidation. Metabolites are displayed on the left and bottom. Red font indicates lipids and their metabolites; blue font indicates amino acids and their derived metabolites. Both dot size and color intensity represent correlation values; red indicates positive correlation, blue indicates negative correlation, and the closer the absolute value is to 1, the stronger the positive or negative correlation. (**C**) AM48h vs. CK48h. (**D**) AM96h vs. CK96h. (**E**,**F**) Differential abundance score (DA score) of KEGG metabolic pathways. (**E**) AM48h vs. CK48h. (**F**) AM96h vs. CK96h. The KEGG pathway enrichment analysis is based on the hypergeometric distribution. Significance levels are indicated as: *, *p* < 0.05; **, *p* < 0.01; ***, *p* < 0.001.

To assess the effects of silk spinning deficiency on fat body metabolism, untargeted metabolomic analysis was performed on fat body tissues at 48 and 96 h after modeling. Partial least squares-discriminant analysis (PLS-DA) revealed distinct metabolic profiles among the AM48h, CK48h, AM96h, and CK96h groups (Figure 4A). At 48 h after modeling, 534 DAMs were identified between the AM and CK groups, with 168 upregulated and 366 downregulated DAMs in the AM group relative to the CK group. At 96 h, 760 DAMs were identified between the two groups, with 262 upregulated and 498 downregulated DAMs in the AM group (Figure 4B).

Correlation analysis of DAMs showed that at 48 h after modeling, oleoyl-L-carnitine was positively correlated with L-tyrosine and L-isoleucine, but negatively correlated with L-(+)-arginine and the phospholipid metabolites PC (16:2/20:4), PE (O-8:0/8:0), and PS (17:1/19:2) (Figure 4C). At 96 h after modeling, L-isoleucine and L-methionine were negatively correlated with L-tyrosine and O-tyrosine (Figure 4D), suggesting that silk spinning deficiency affects the pattern of amino acid accumulation pattern in the fat body over time.

Differential abundance analysis of KEGG metabolic pathways showed that the significantly upregulated pathways at 48 h after modeling mainly involved the Citrate cycle (TCA cycle), Fatty acid elongation, Fatty acid degradation, Cysteine and methionine metabolism, and Tryptophan metabolism (Figure 4E). At 96 h after modeling, Tyrosine metabolism, Alanine, aspartate and glutamate metabolism, and Arginine biosynthesis were significantly upregulated, whereas Tryptophan metabolism, Fatty acid degradation, FoxO signaling pathway, and Cysteine and methionine metabolism were significantly downregulated in the AM group (Figure 4F). These results indicate that silk spinning deficiency causes remarkable changes in amino acid and lipid metabolism in the fat body.

**Figure 5:**
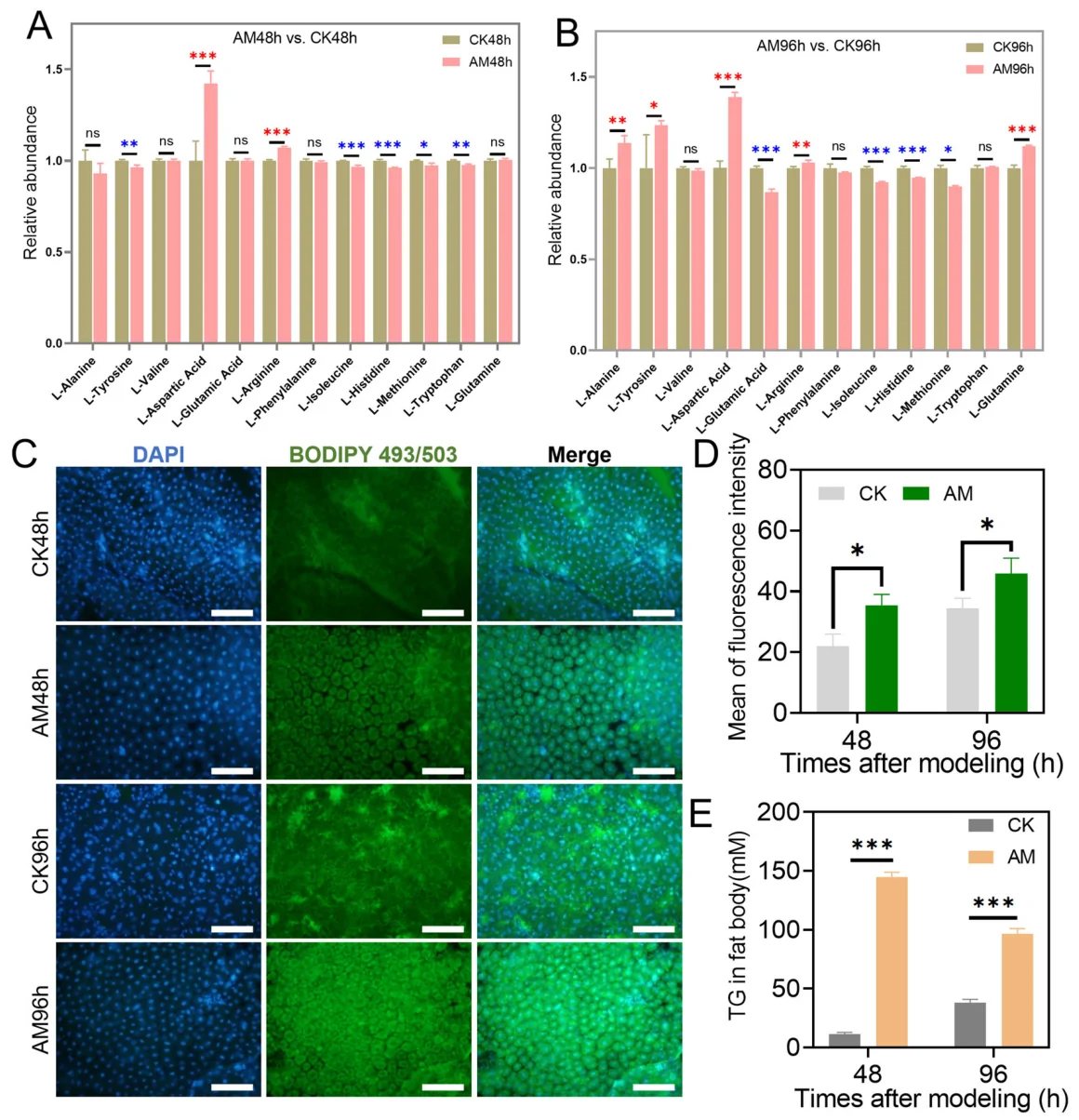
Abnormal lipid accumulation in the fat body of silkworms in the silk spinning deficiency model. (**A**,**B**) Changes in amino acid content in the metabolomic. N = 5. (**A**) AM48h vs. CK48h. (**B**) AM96h vs. CK96h. Red indicates higher levels in the AM group than in the CK group, and blue indicates lower levels in the AM group than in the CK group. (**C**,**D**) Lipid droplet content in the fat body. N = 3. (**E**) TG content in the fat body. N = 3. Significance levels are indicated as: *, *p* < 0.05; **, *p* < 0.01; ***, *p* < 0.001; ns, *p* > 0.05.

Further analysis of free amino acid abundance in the fat body showed that at 48 h after modeling, aspartate and arginine abundances were significantly higher in the AM group than in the CK group, whereas tyrosine, isoleucine, histidine, methionine, and tryptophan abundances were significantly lower in the AM group (Figure 5A). At 96 h after modeling, in addition to aspartate and arginine, glutamate and the silk protein substrates alanine and tyrosine were also significantly more abundant in the AM group than in the CK group (Figure 5B).

To determine the impact of silk spinning deficiency lipid metabolism in the fat body, lipid droplets were stained at 48 and 96 h after modeling. Lipid droplet abundance was significantly higher in the AM group than in the CK group at both time points (Figure 5C,D). Because lipids are mainly stored as TG in the fat body, we performed TG measurements, which revealed that fat body TG content was significantly higher in the AM group than in the CK group at 48 and 96 h after modeling (Figure 5E).

In combination with fat body transcriptomic analysis, the transcript level of *Lsd2*, a gene related to lipid droplet stability, was significantly higher in the AM group than in the CK group at both 48 and 96 h after modeling (Figure S2A). *AGPAT*, a key enzyme in triglyceride synthesis, was significantly upregulated at 48 h after modeling (Figure S2B). Meanwhile, *FAS*, the sole core enzyme for endogenous fatty acid synthesis in the fat body, showed no significant difference compared with the CK group at 48 h after modeling but was significantly upregulated at 96 h (Figure S2C).

**Figure 6:**
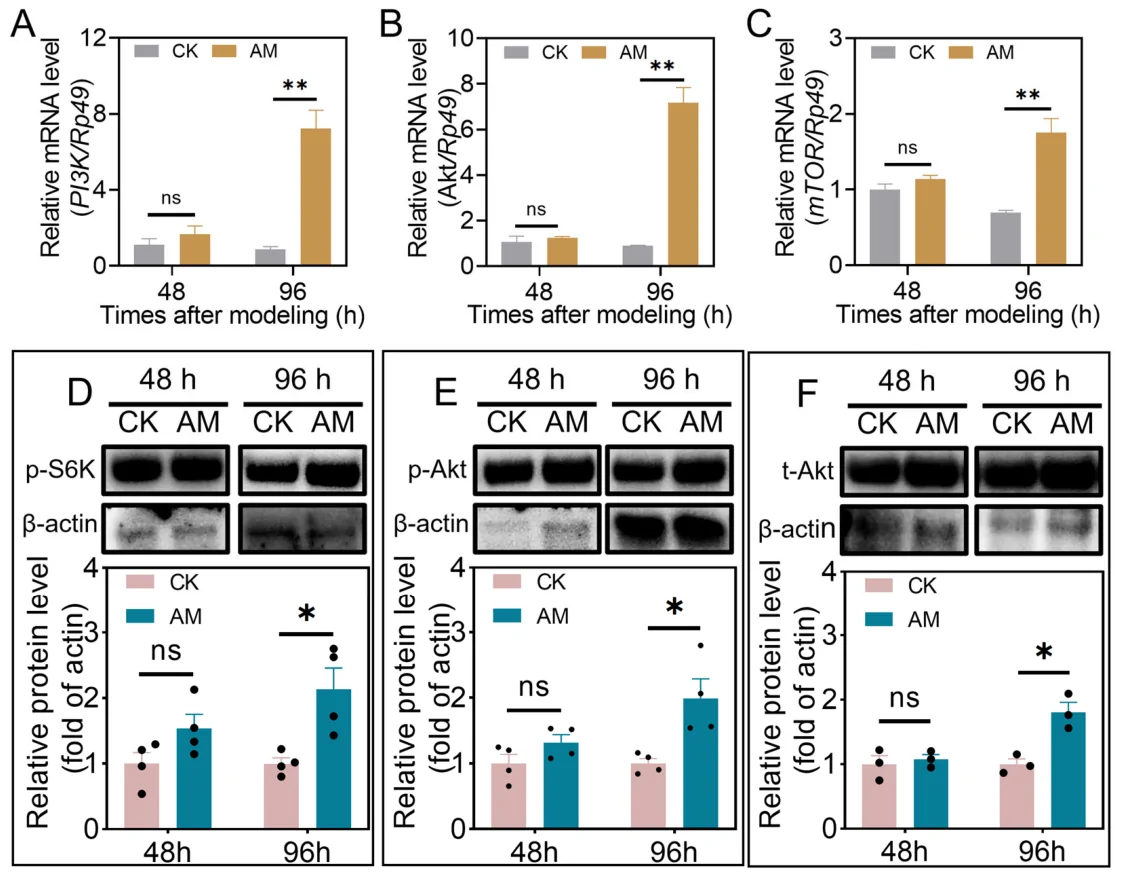
Elevated IIS/TOR pathway expression in the fat body of silkworms with silk spinning deficiency. (**A**–**C**) Transcript levels of IIS/TOR pathway-related genes. N = 3. (**A**) *PI3K*. (**B**) *Akt*. (**C**) *mTOR*. (**D**–**F**) Protein levels of insulin signaling pathway-related components. (**D**) Phosphorylated S6K protein, p-S6K. N = 4. (**E**) phosphorylated Akt protein, p-Akt. N = 4. (**F**) total Akt protein, t-Akt. N = 3. Significance levels are indicated as: *, *p* < 0.05; **, *p* < 0.01; ns, *p* > 0.05.

Moreover, the transcript levels of insulin signaling pathway-related genes regulating lipid metabolism, including *PI3K*, *Akt*, and *mTOR*, did not differ significantly at 48 h after modeling but were significantly higher than those in the CK group at 96 h (Figure 6A–C). Protein levels were further examined, and the level of p-S6K, a positive downstream regulator of mTOR, was significantly increased at 96 h, consistent with the transcript level of *mTOR* (Figure 6D). In the AM group, phosphorylated Akt (p-Akt) and total Akt (t-Akt) levels in the fat body at 48 h after modeling showed no significant differences when compared with those in the CK group, whereas both were significantly increased at 96 h (Figure 6E,F).

These results suggest that silk spinning deficiency may promote excessive lipid accumulation in the fat body by upregulating the expression of genes related to lipid synthesis and the IIS/TOR nutrient-sensing signaling pathway.

## 4. Discussion

Metamorphic development in holometabolous insects involves larval tissue degeneration and adult tissue remodeling [16,32,33]. After spinning is completed in the silkworm, silk gland degeneration and fat body dissociation and remodeling are initiated, and nutrients released during silk gland degeneration can enter the hemolymph and contribute to nutrient redistribution during metamorphosis [3,5]. In this study, silkworms with silk spinning deficiency showed delayed silk gland degeneration and a concomitant delay in the initiation of fat body dissociation, accompanied by extensive transcriptional and metabolic changes. These results suggest that the effects of silk spinning deficiency are not restricted to the silk gland but may involve global changes in the endocrine and nutritional environment during metamorphosis, thereby affecting the coordination of developmental timing among tissues.

Silk spinning deficiency leads to abnormal pupation in silkworms, and CK and AM may be at different developmental stages at the same time point. Morphological observation of the fat body showed that both CK48 h and AM96 h were at the dissociation initiation stage, with similar fat body morphological characteristics. Further comparison of lipid droplets/TG (Figure S5), key genes of amino acid catabolism (Figure S6), key glycolysis genesbetween CK 48 h and AM 96 h indicated that silk spinning deficiency causes developmental delay accompanied by metabolic reprogramming.

During metamorphosis, JH directly represses Mmp1 transcription through Kr-h1; after the decline in JH, the 20E pulse activates Mmp expression through EcR/Bftz-F1, and the temporal coordination of the two drives dissociation [34,35,36]. In the AM group, JH signaling was downregulated (Figure S3A–C) and 20E pathway genes were upregulated (Figure S3D–H), yet dissociation was still delayed, indicating that relief of JH repression was not effectively translated into dissociation execution. The exogenous 20E rescue experiment provides key support for this interpretation: in the silk protein retention model, injection of 20E alleviated the inhibition of fat body remodeling and improved pupation progression, demonstrating that the fat body in this state retains the ability to respond to 20E, and that the limiting factor is insufficient endogenous 20E signaling rather than loss of the response mechanism [18]. In addition, hemolymph 20E titers were significantly reduced in this model, and the 20E-Vg/VgR pathway was suppressed, which could be restored by exogenous 20E [37]. Therefore, although the decline in JH in the AM group partially relieved the repression of Mmp1, the 20E titer/signaling intensity did not reach the threshold required to trigger dissociation, which may be the direct rate-limiting factor for the delayed dissociation. The nutrient-excess state may further reinforce this limitation. In the fat body, 20E signaling interacts with the IIS/TOR pathway: 20E can antagonize TOR/insulin signaling, while persistent nutrient signaling can also affect the initiation and efficiency of 20E responses. The fat body is in a state of persistent nutrient excess and lipid storage, and this metabolic environment may lower 20E titers, delay the timing of its pulse, or interfere with the full activation of downstream pro-dissociation programs of 20E. In other words, the delayed dissociation should not be simply attributed to “JH remaining high” or “insufficient 20E,” but is more likely the combined result of insufficient relief of JH repression and insufficient 20E drive under interference from the nutritional state.

Fat body dissociation and remodeling during metamorphosis involve dramatic morphological and functional changes. Silk spinning deficiency markedly altered gene expression patterns, mainly in carbohydrate, amino acid, and lipid metabolism, indicating that the fat body metabolic response to silk spinning deficiency intensifies over time. During normal metamorphosis, the fat body undergoes stage-specific metabolic switches to meet nutrient storage, mobilization, and redistribution demands [19,38]. However, the differences between the AM and CK groups continued to widen over time, suggesting additional metabolic adaptations beyond developmental delay. The pronounced directional shift in glucose metabolism suggests that pyruvate may be diverted to lactate production rather than oxidative phosphorylation for energy generation. Previous studies have shown that silkworms reduce energy dependence on glycolysis and oxidative phosphorylation during metamorphosis and instead use amino acid reserves for energy through transdeamination [39]. These metabolic changes suggest that the fat body neither effectively completed energy mobilization nor shifted to the reserve metabolism required for the pupal stage according to the normal program.

Disruption of amino acid metabolism more directly reflects the shift in amino acid flux caused by silk spinning deficiency. Differential expression of metabolic pathways for the major amino acid constituents of silk proteins induced by silk spinning deficiency suggests that the amino acid catabolism and transamination/deamination networks in the fat body are reconfigured [40,41]. The dynamic changes in free amino acid abundance indicate that sustained accumulation of substrate amino acids in the hemolymph caused by silk spinning deficiency may drive excessive amino acid influx into the fat body via a concentration gradient, thereby leading to metabolic overload in the fat body [30,42,43].

Amino acid and lipid metabolism are closely interconnected in the insect fat body [44,45]. In this study, at 96 h after modeling, transcript levels of amino acid catabolism-related genes, including alanine aminotransferase, glutaminase, arginase, tryptophan 2,3-dioxygenase, and aspartate decarboxylase, were significantly upregulated in the AM group. This suggests that silk spinning deficiency may expose the fat body to persistent nutrient excess signals, overactivating amino acid catabolism and shunting its degradation products (carbon skeletons) into lipid synthesis pathways. Lipid droplet staining and triglyceride measurements further supported this inference. Moreover, lipid storage- and synthesis-related genes showed varying degrees of transcriptional upregulation in the AM group (Figure S2A–C). We speculate that silk spinning deficiency causes massive accumulation of amino acids that serve as silk protein substrates. The fat body converts the carbon skeletons of these excess amino acids into fatty acids and triglycerides for storage while upregulating Lsd2 to inhibit lipid droplet degradation, ultimately leading to excessive lipid accumulation. Such excessive lipid accumulation may further delay the dissociation process by affecting the physical properties and signal transduction environment of fat body cells [46,47].

The hemolymph nutritional environment is an upstream link connecting silk gland dysfunction and fat body metabolic reprogramming. Previous studies have shown that hemolymph total protein increased significantly from 48 h after modeling, followed by increases in free threonine, arginine, and tryptophan; at 96 h, the major silk protein amino acids alanine, serine, and tyrosine also accumulated significantly [30,31]. These changes in hemolymph nutrition correspond to the lipid accumulation at 48 h, the upregulation of amino acid catabolism genes at 96 h, and IIS/TOR activation at 96 h observed in this study. These findings support that hemolymph nutrient accumulation is an important upstream signal associated with fat body metabolic reprogramming.

IIS/TOR signaling is a core pathway regulating nutrient sensing, growth, and metabolism in insects [48,49,50]. Inhibition of the insulin receptor (InR) reduces fat body triglyceride content, decreases lipid droplet diameter, and suppresses de novo lipogenesis [51], whereas enhanced insulin signaling pathway activity promotes fat body cell enlargement and increases triglyceride levels [52,53], indicating that the IIS pathway positively regulates lipid accumulation in the insect fat body. In naked pupae produced by silk gland dysfunction that inhibits fibroin and sericin gene expression, the fat body maintains high lipid and carbohydrate reserves, accompanied by upregulated IIS/TOR signaling [21]. In this study, silk protein synthesis was normal, but silk spinning was blocked, leading to nutrient excess in the circulatory system. Under persistent nutrient excess signals, the fat body activated the IIS/TOR pathway and stored excess carbon sources in the form of lipids. Notably, IIS/TOR signaling activation (96 h) occurred later than the onset of lipid accumulation (48 h), and the upregulation of Lsd2 and AGPAT preceded IIS/TOR signaling activation, suggesting that the initiation of lipid accumulation may be driven by a passive increase in amino acid substrate supply, while IIS/TOR pathway activation further enhances and maintains lipid accumulation [54]. In addition, enrichment of the PPAR signaling pathway may cooperate with IIS/TOR to sustain the trend of lipid accumulation [55,56].

Amino acids released by silk gland degeneration not only serve as energy substrates but may also act as nutritional signals participating in the regulation of the fat body reproductive nutritional program [57,58,59]. Previous studies have suggested that amino acids released by silk gland metabolism can induce GATA and other nutrient-sensitive transcription factors and promote the synthesis of vitellogenin (Vg) and ovalbumin [21,26]. In this study, the abundances of free amino acids such as glutamate, alanine, and tyrosine in the fat body of the AM group were increased at 96 h (Figure 5B), genes related to amino acid catabolism were significantly upregulated (Figure 3D–H), and IIS/TOR signaling was activated at 96 h (Figure 6), suggesting that the fat body was in a state of persistent overnutrition and that the GATA-Vg ovalbumin program might be initiated. However, the expression levels of Vg and GATAβ in the CK group were higher than those in the AM group at 48 h, and the differences in expression of these two genes between the two groups were no longer significant after 96 h, indicating that directing excess carbon sources to reproductive nutrition may not be the highest priority in the fat body. In this model, silk gland development and silk protein synthesis were normal, and only spinning was blocked; silk gland degeneration was significantly delayed (Figure S1C). The sequence of amino acid release differed from that of the cocoon-free model generated by inhibiting silk protein synthesis. Moreover, lipid accumulation appeared at 48 h, earlier than IIS/TOR activation (Figure 5C–E and Figure 6), indicating that excess carbon sources were first directed to lipid storage. Therefore, activation of the GATA-Vg ovalbumin axis cannot be inferred from amino acid abundance alone; it may reflect delayed activation, uncoupling from lipid storage, or asynchrony across tissues.

In summary, silk spinning deficiency disrupts nutrient redistribution during metamorphosis, leading to metabolic abnormalities in the fat body. During normal metamorphosis, spinning mediates the concentrated export of proteins and amino acids from late larvae. When silk spinning is blocked, these resources cannot be exported through normal routes, and the fat body alters its utilization patterns of carbohydrates and amino acids, with enhanced amino acid catabolism and lipid synthesis and sustained triglyceride accumulation. Under persistent nutrient excess, the IIS/TOR nutrient-sensing pathway is activated, further sustaining lipid synthesis and storage.

## 5. Conclusions

Silk spinning deficiency delays the initiation of fat body dissociation and impairs the remodeling process during metamorphosis in the silkworm. Integrated transcriptomic and metabolomic analyses indicate that silk spinning deficiency induces transcriptional reprogramming of metabolism-related genes in the fat body, leading to continuous conversion of excess amino acids into lipids and activation of IIS/TOR nutrient-sensing signaling, which sustains excessive lipid accumulation in the fat body.

## Data Availability

The transcriptomics and metabolomics data presented in this study are included in the article and its Supplementary Materials. All sequencing data are available through the NCBI Sequence Read Archive under the accession number PRJNA1557045. Metabolomics data are available through the China National Center for Bioinformation/National Genomics Data Center (CNCB-NGDC) under BioProject PRJCA078949 and OMIX identifier OMIX023121. Further inquiries may be directed to the corresponding author.

## Supplementary Materials

The following supporting information can be downloaded at https://www.mdpi.com/article/10.3390/biology15212025/s1, Figure S1. Modeling and morphological development of silkworm with silk spinning deficiency model; Figure S2. Changes in transcription levels of genes related to lipid storage and synthesis; Figure S3. Statistical analysis of gene expression levels in JH and 20E signaling; Figure S4. KEGG pathway enrichment analysis of CK48h vs. AM96h; Figure S5. Difference in lipid accumulation between CK48h and AM96h; Figure S6. Difference in gene expression between CK48h and AM96h; Table S1. Gene-specific primers used in qRT-PCR; Table S2. AM48h_vs_AM96h.deseq2.annot; Table S3. CK48h_vs_AM48h.deseq2.annot; Table S4. CK96h_vs_AM96h.deseq2.annot; Table S5. CK48h_vs_CK96h.deseq2.annot; Table S6. CK48h_vs_AM96h.deseq2.annot; Table S7. CK48h_vs_AM48h_metabolites; Table S8. CK96h_vs_AM96h_metabolites.

## Abbreviations

The following abbreviations are used in this manuscript:

AM	silk spinning deficiency animal model
CK	control group
qRT-PCR	quantitative reverse transcription polymerase chain reaction
TPM	transcripts per million
DEG	differentially expressed gene
DAM	differential accumulation metabolite
TG	triglyceride
SRd	start of fat body remodeling
Rd	fat body remodeling process
IS	intercellular space
CRd	completion of fat body remodeling
Mmp1	matrix metalloproteinase 1
Mmp3	matrix metalloproteinase 3
MMPs	matrix metalloproteinases
AdamTS	a disintegrin and metalloproteinase with thrombospondin motifs 1
AdamTSL	a disintegrin and metalloproteinase with thrombospondin motifs like
TIMs	tissue inhibitor of metalloproteinases
Alt	alanine aminotransferase 1
Glu	glutaminase
Arg	arginase
Tdo2	tryptophan 2,3-dioxygenase
Adc	aspartate decarboxylase
Shmt	serine hydroxymethyltransferase
Agxt	serine-pyruvate aminotransferase
Hk	hexokinase type 2
Ldh	lactate dehydrogenase
Cs	citrate synthase
Idh	isocitrate dehydrogenase
6PGD	6-phosphogluconate dehydrogenase
Pepck	mitochondrial phosphoenolpyruvate carboxykinase
Fbp	fructose-1,6-bisphosphatase
p-S6K	Phosphorylated S6K protein
p-Akt	phosphorylated Akt protein
t-Akt	total Akt protein
JH	juvenile hormone
20E	20-hydroxyecdysone
InR	insulin receptor
Lsd2	perilipin
AGPAT	1-acylglycerol-3-phosphate O-acyltransferase
FAS	Fatty acid synthase
