# Supplementary material for: Silk Spinning Deficiency Causes Delayed Fat Body Dissociation Accompanied by Lipid Metabolic Reprogramming in Bombyx mori
Source: Biology (Basel). 2026 Nov 6;15(21):2025. doi: 10.3390/biology15212025 (PMC13762255; doi:10.3390/biology15212025)
Supplement: Supplementary file 1 [file biology-15-02025-s001.zip › Supplemental Materials.pdf]

Silk Spinning Deficiency Causes Delayed Fat Body Dissociation Accompanied by Lipid Metabolic Reprogramming in *Bombyx mori*

Ruji Peng [1,2,†], Xiaoning Sun [1,2,†], Cheng Luo [1,2], Jianghan Du [1,2], Yanhong Jiang [1,2], Yanghu Sima [1,2], Guang Wang [1,2,*] and Shiqing Xu [1,2,*]

[1] School of Life Sciences, Suzhou Medical College, Soochow University, Suzhou 215123, China
[2] Institute of Agricultural Biotechnology & Ecology (IABE), Soochow University, Suzhou 215123, China
* Correspondence: gwang1994@suda.edu.cn (G.W.); szsqxu@suda.edu.cn (S.X.); Tel.: +86-512-65880185 (S.X.)
† These authors contributed equally to this work.

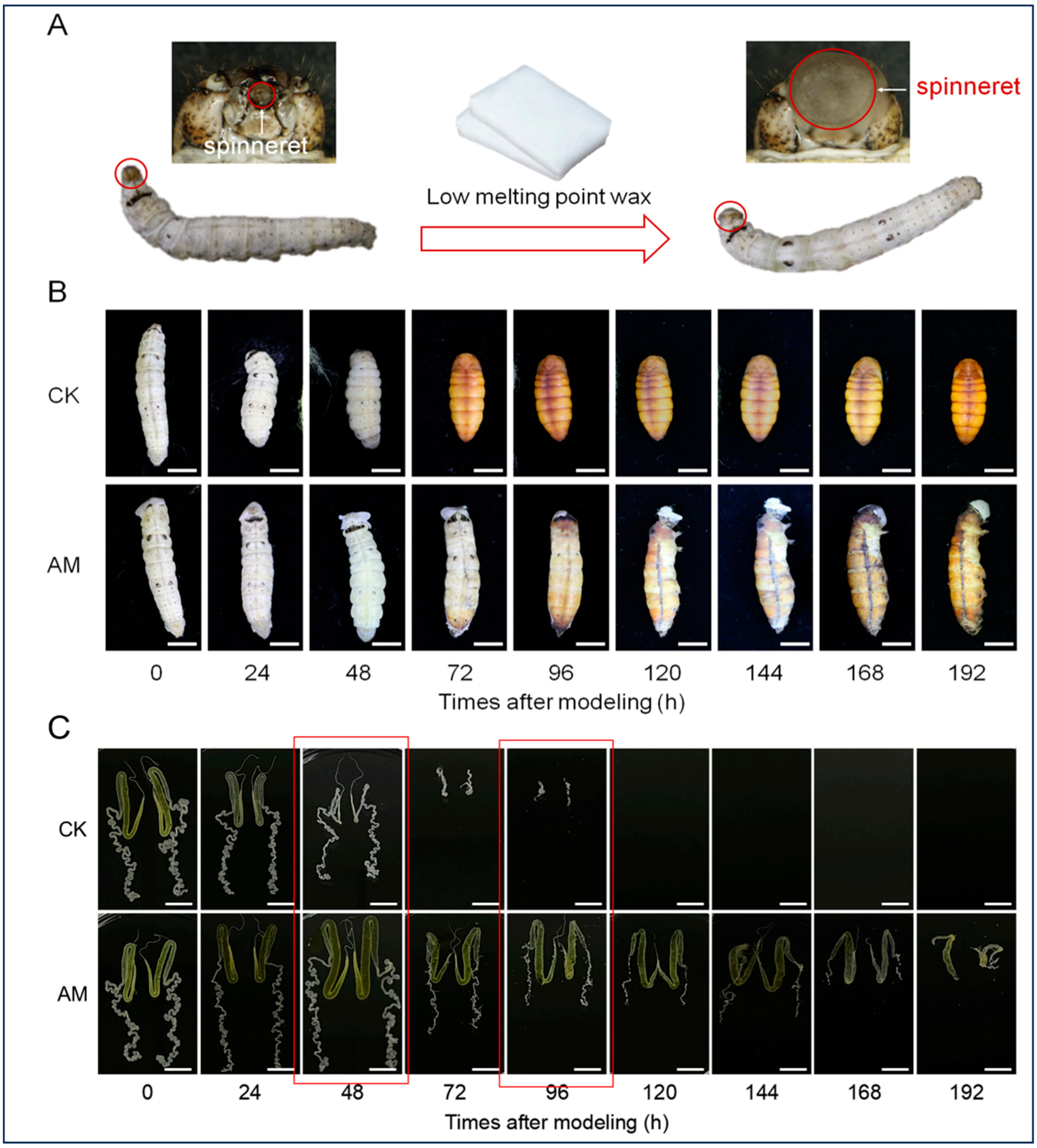

Figure S1. Modeling and morphological development of silkworm with silk spinning deficiency model. (A) Modeling method. (B) The morphological development map of the silk spinning deficiency model group (AM) and control group (CK). The silkworm in the CK group pupated normally; the silkworm in the AM group was hypoplastic and half-pupated. (C) Morphological changes of the silk gland in *Bombyx mori*. Scale = 1 cm.

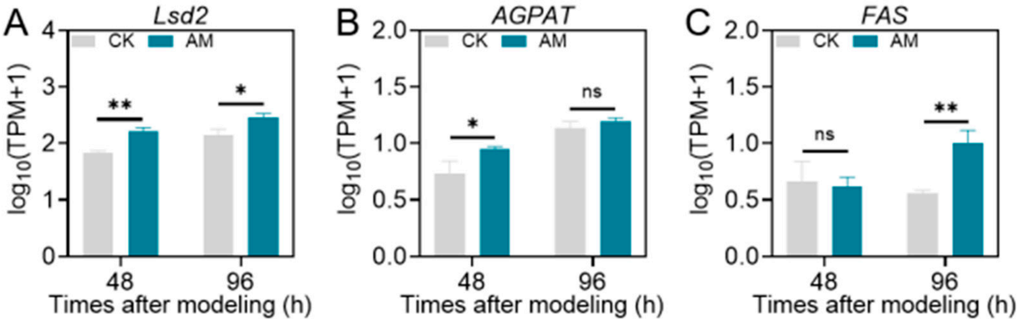

Figure S2. Changes in transcription levels of genes related to lipid storage and synthesis. A, *Lsd2*, perilipin. B, *AGPAT*, 1-acylglycerol-3-phosphate O-acyltransferase. C, *FAS*, fatty acid synthase.Three independent biological replicates were analyzed. Significance levels are indicated as: *, $P < 0.05$; **, $P < 0.01$; ***, $P < 0.001$; ns, $P > 0.05$. n = 3.

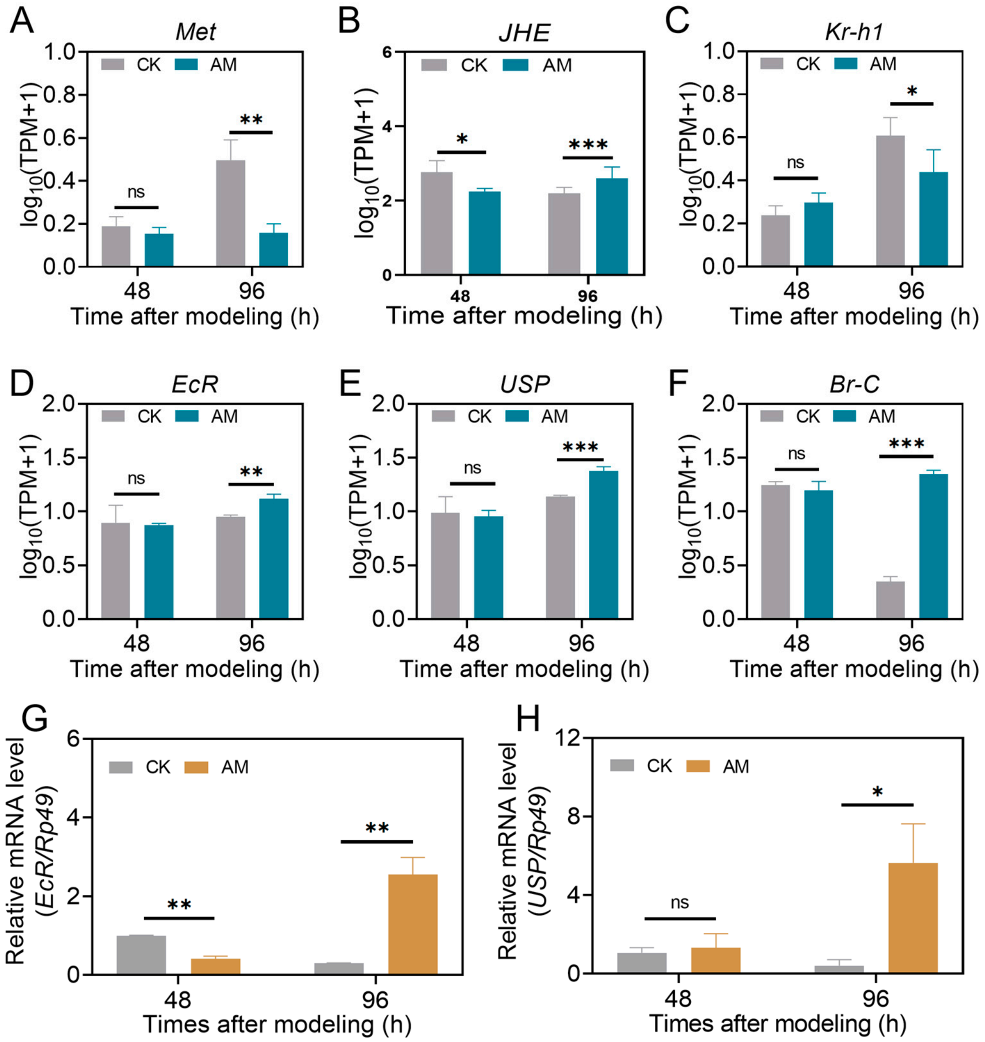

Figure S3. Statistical analysis of gene expression levels in JH and 20E signaling . (A-C) Changes in gene transcription levels of JH signaling. A, *Met*, juvenile hormone resistance protein II. B, *JHE*, juvenile hormone esterase 1. C, *Kr-h1*, Kruppel homolog 1. (D-F) Changes in gene transcription levels of 20E signaling. D, *EcR*, ecdysone receptor. E, *USP*, RXR type hormone receptor. F, *Br-C*, broad-complex. (G-H) mRNA levels of 20E signaling. G, *EcR*. H, *USP*. Rp49 was used as the internal reference gene. Significance levels are indicated as: *, $P < 0.05$; **, $P < 0.01$; ***, $P < 0.001$; ns, $P > 0.05$. N = 3. Three independent biological replicates were analyzed.

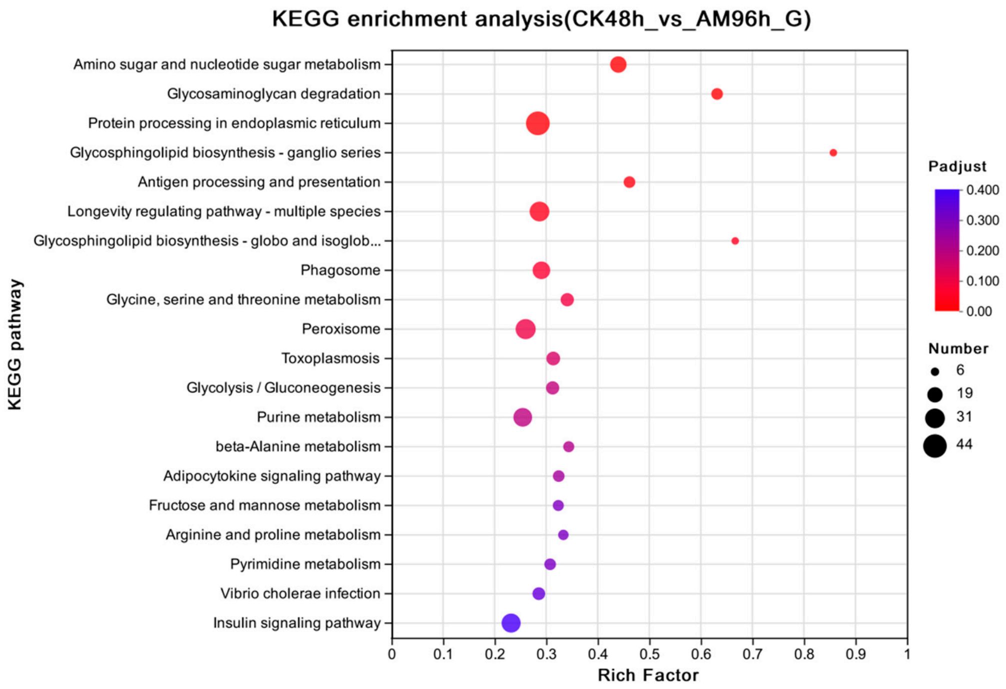

Figure S4. KEGG pathway enrichment analysis of CK48h vs. AM96h.

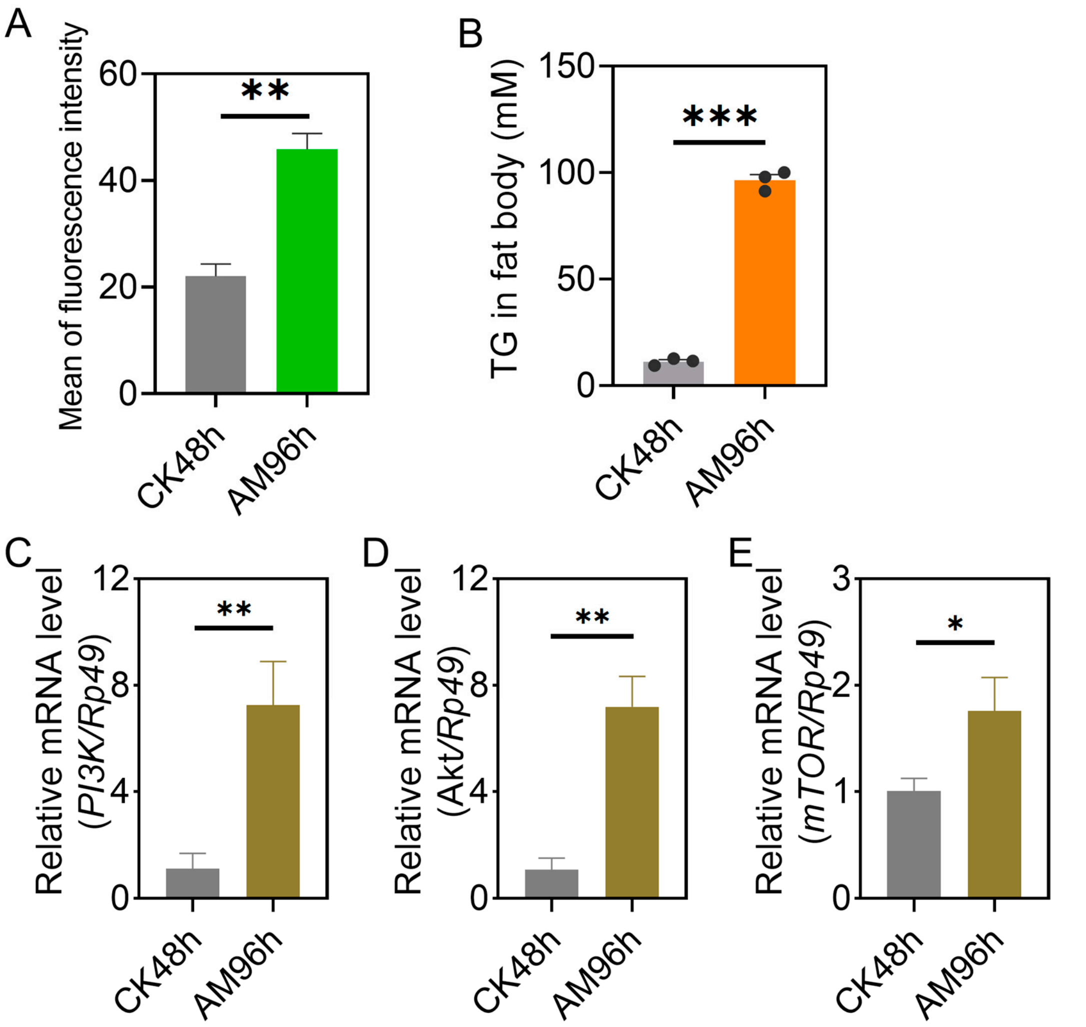

Figure S5. Difference in lipid accumulation between CK48h and AM96h. (A) Lipid droplet content in the fat body. N = 3. (B) TG content in the fat body. N = 3. (C-E) Transcript levels of IIS/TOR pathway-related genes. N = 3. C, *PI3K*. D, *Ak*t. E, *mTOR*. *Rp49* was used as the internal reference gene. Three independent biological replicates were analyzed. Significance levels are indicated as: *, $P < 0.05$; **, $P < 0.01$; ***, $P < 0.001$; ns, $P > 0.05$. N= 3.

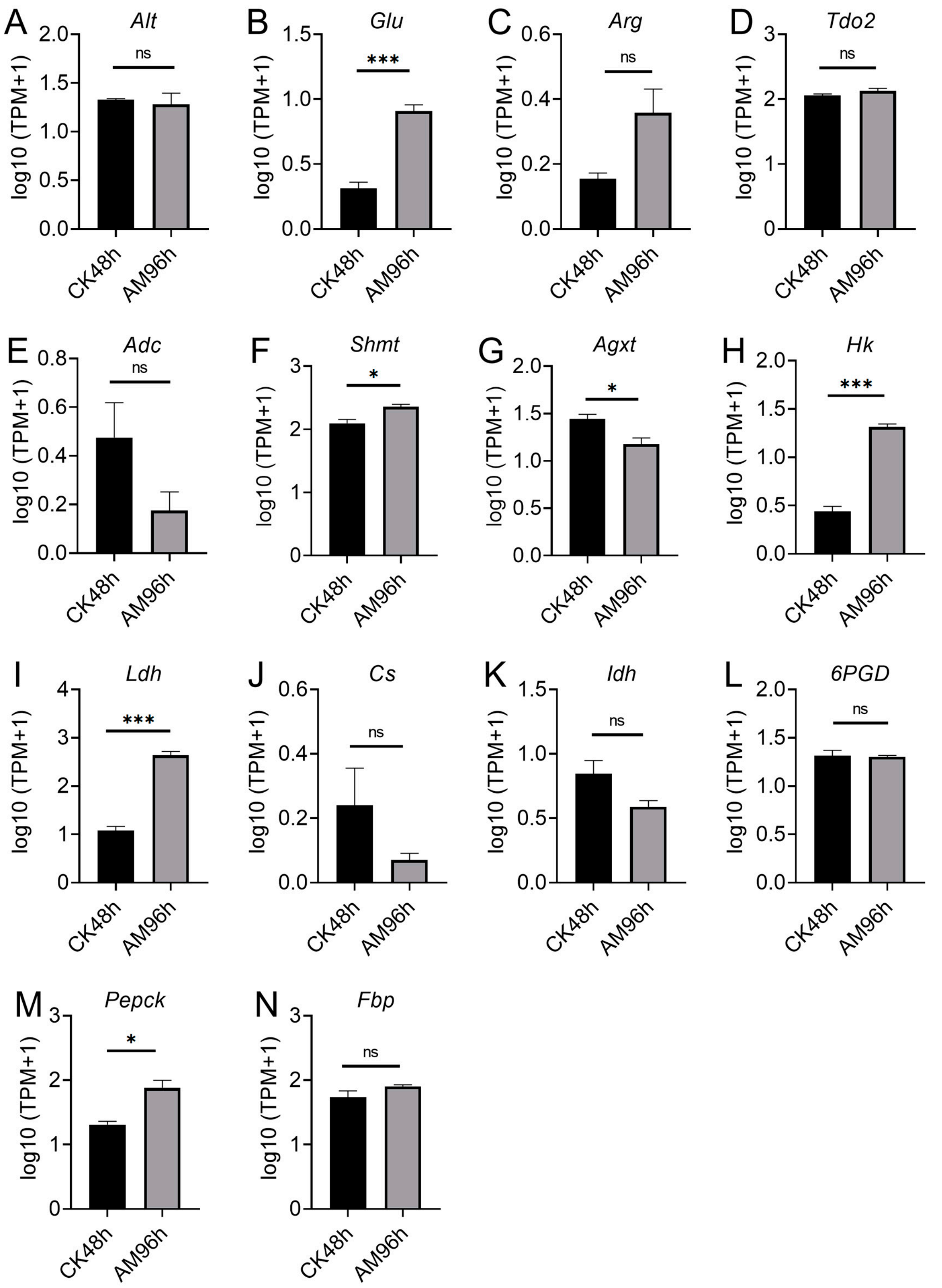

Figure S6. Difference in gene expression between CK48h and AM96h. Three independent biological replicates were analyzd. Significance levels are indicated as: *, $P < 0.05$; **, $P < 0.01$; ***, $P < 0.001$; ns, $P > 0.05$. n = 3.

Table S1. Gene-specific primers used in qRT-PCR

Genes	Primers	Forward (5′ to 3′)	Reverse (5′ to 3′)
matrix metalloprotein ase 1	Mmp1	AAGCCTTTGTCTAACTGGGATGG	GCGGGAACTGAGGGTTGTCT
matrix metalloprotein ase 3	Mmp3	CCGGAGTTGGCTACACTTACAC	CCTTTCTATGCGATCCTTGCT
A disintegrin and metalloprotein ase with thrombospon din motifs 1	AdamTS	GGGTCTTTGGCTTGAACT	AATTATGTCCTTGTCCTCGTA
A disintegrin and metalloprotein ase with thrombospon din motifs like	AdamTS-L	TGAGAAGGGGTTCAGATG	CTGAGCGTCCAGGGTAA
phosphatidylino sitol 3-kinase	PI3K	CGGAACGCAGAAGACTTACATC	TATCCCGCCTAAACAGACAACC
target of rapamycin	mTOR	ATGAAAATTTCTGACAAAGGC	TCCATAGCTACACAAACTAAGC
serine/threoni ne protein kinase	Akt	TCCCGCCGTAGATTGGT	GATGAGCCCGAACAGCAC
ecdysone receptor	EcR	CCACGATGCCTTTACCAATG	GTCGAGGTGCAGGACCTTTC
RXR type hormone receptor	USP	ATGTCAGTGGCGAAGAAAGA	ATCCAGCGAACAGTCAACAG
RpL32	Rp49	CAGGCGGTTCAAGGGTCAATAC	TACGGAATCCATTTGGGAGCAT
